# The London County Council

**Published:** 1915-11-13

**Authors:** 


### THE LONDON COUNTY COUNCIL.

### LYING-IN HOMES.

At the London County Council on Tuesday an emergency report was brought forward by the General Purposes Committee with reference to lying-in homes. The Committee has secured powers to supervise these institutions, and has the power to delegate such powers to the Borough Councils. The report stated that the Committee had not yet had ample opportunity of considering whether or not the power should be exercised to its full extent, but considered that the Council should itself undertake the initial registration of such establishments, and that it should take all steps necessary to this end, including, if necessary, preliminary inspection. The Committee was further considering the question of delegation of the duty of inspection, and proceeded to state :

> The preamble of the Act expresses the grounds upon which powers under Part IV. were sought as to (1) the prevention of immorality, and (2) the interests of the health of women received in lying-in homes for the purposes of childbirth. In this connection, however, we have to draw attention to the fact that not only is the dealing with lying-in homes closely associated with the administration of the Infant Life Protection provisions of the Children Act, but also with that of the Midwives Act. Were it not for their peculiar statutory position the Midwives Act Committee would be the proper Committee to undertake the administration of this part of the General Powers Act as well as of the work under the other Acts mentioned. The Infant Life Protection provisions of the Children Act are now administered by the Public Control Committee. The principles which are to govern the Council in refusing registration are prescribed by Section 17 as (1) the bad character of the keeper of the home; (2) the unsuitableness of the premises and equipment; and (3) the use of the premises for immoral purposes. On the balance it appears to us that the Public Control Committee are the appropriate Committee.

Mrs. W. Phipps urged that the work would be done better by the Midwives Act Committee, but Mr. W. C. Johnson remarked that it would be grotesque to send round lady doctors to make the inquiries that were necessary, adding that the Committee wished to ascertain the character of some of these premises. In the end the Council adopted the report, which means that the work will be done by the Public Control Committee.

### MASSAGE ESTABLISHMENTS.

Henceforth establishments for massage or special treatment come under the supervision of the L.C.C., which on Tuesday authorised Miss K. M. M. McGuinness and Mr. P. McIntyre, inspectors of the Public Control Department, to enter and inspect any premises used, or believed to be used, for the purposes of an establishment for massage or special treatment. From a statement circulated by the Borough Councils Joint Committee, it seems that, owing to deletions from the L.C.C. Bill in the House of Lords, only a comparatively few premises are affected, and in these cases the County Council did not insert a clause for delegating its powers to the Borough Councils.

